# Foster Dams Rear Fighters: Strain-Specific Effects of Within-Strain Fostering on Aggressive Behavior in Male Mice

**DOI:** 10.1371/journal.pone.0075037

**Published:** 2013-09-10

**Authors:** Kimberly H. Cox, Nina L. T. So, Emilie F. Rissman

**Affiliations:** Department of Biochemistry and Molecular Genetics and Graduate Program in Neuroscience, University of Virginia School of Medicine, Charlottesville, Virginia, United States of America; Université de Bordeaux and Centre National de la Recherche Scientifique, France

## Abstract

It is well known that genes and environment interact to produce behavioral phenotypes. One environmental factor with long-term effects on gene transcription and behavior is maternal care. A classic paradigm for examining maternal care and genetic interactions is to foster pups of one genetic strain to dams of a different strain ("between-strain fostering"). In addition, fostering to a dam of the same strain ("within-strain fostering") is used to reduce indirect effects, via behavioral changes in the dams, of gestation treatments on offspring. Using within-and between-strain fostering we examined the contributions of genetics/prenatal environment, maternal care, and the effects of fostering *per se*, on adult aggressive behavior in two inbred mouse strains, C57BL/6J (B6) and DBA/2J (DBA). We hypothesized that males reared by dams of the more aggressive DBA strain would attack intruders faster than those reared by B6 dams. Surprisingly, we found that both methods of fostering enhanced aggressive behavior, but only in B6 mice. Since all the B6 offspring are genetically identical, we asked if maternal behavior of B6 dams was affected by the relatedness of their pups. In fact, B6 dams caring for foster B6 pups displayed significantly reduced maternal behaviors. Finally, we measured vasopressin and corticotrophin releasing hormone mRNA in the amygdalae of adult B6 males reared by foster or biological dams. Both genes correlated with aggressive behavior in within-strain fostered B6 mice, but not in mice reared by their biological dams. In sum, we have demonstrated in inbred laboratory mice, that dams behave differently when rearing their own newborn pups versus pups from another dam of the same strain. These differences in maternal care affect aggression in the male offspring and transcription of *Avp* and *Crh* in the brain. It is likely that rearing by foster dams has additional effects and implications for other species.

## Introduction

The last two decades have witnessed an increased interest in the field of maternal behavior, as we now know that natural variation in the amount of maternal care provided to infants dictates many of their adult behaviors [1], [2], [3]. In addition, short-term maternal separation also influences behaviors of adult offspring [4], [5], [6]. The neuronal mechanisms that underlie these changes in behaviors include altered transcription of critical genes in specific brain regions [2], [5], [7], [8]. In addition, some gene transcription differences correlate with levels of DNA methylation of the affected gene promoters [3], [9], [10], [11]. Thus, there is substantial evidence that maternal care and the early environment result in epigenetic modifications that have long-term effects on behavior (reviewed in [12], [13]).

Because the maternal environment can have powerful actions on offspring, it should be treated as a dependent variable in experiments where the behavior of the dam may be influenced by treatment. "Within-strain" fostering, whereby pups are removed from their dam at birth and reared by dams with the same genetic background, is a common method for providing consistent maternal care [14], [15], [16]. On the other hand, "between strain" fostering is also commonly used to assess the independent actions of genetics and prenatal environment versus the postnatal environment in studies examining behavioral differences between rodent strains [17], [18], [19]. However, even within-strain fostering may also change the behavioral interaction between dams and infants [17], [20], [21].

Here we used both between-strain fostering and within-strain fostering to assess the contributions of genetics/prenatal environment, the postnatal maternal environment, and the effects of fostering *per se*, on adult aggressive behavior in the C57BL/6J (B6) and DBA/2J (DBA) inbred mouse strains. These strains differ from each other in a variety of behavioral and physiological measures [22], [23], [24], but, most significantly for our study, DBA males are more aggressive than B6 males [25]. Importantly, some aspects of maternal care also differ between these two strains; DBA dams take longer to retrieve pups [26], [27] and produce lower quality nests [27], [28] than B6 dams.

There is an extensive literature on the genetic basis strain differences in aggression [29], [30], [31], [32], [33], between the two strains we selected. Some studies examined postnatal maternal contributions in the context of rearing hybrid offspring [34], [35], [36]. However, relatively little attention has been paid to just the post-natal maternal contributions to behavioral differences in these inbred mouse strains. One exception is a study of activity using both within and between-strain fostering of DBA and B6 mice. Both types of fostering altered behavior of B6 males in an open-field, while having no effect on DBA males [22]. While aggression was not measured in this study, in wild deer mice (*Peromyscus*), male offspring from an aggressive species (*P. californicus*) reared by dams of a less aggressive species (*P. leucopus*) exhibited reduced adult agonistic behaviors [18], suggesting that genetic determinants of aggressive behavior are modulated by the maternal environment. Therefore, we hypothesized that the postnatal maternal environment may also contribute to strain differences in B6 and DBA mice. Here we report that the effects of within-strain fostering on B6 male aggression are more significant than the effects of between-strain fostering to DBA dams. Our results also show that moving newborn pups to a non-biological dam significantly alters maternal behavior, while also influencing the adult behavior of offspring and transcription of relevant genes in one brain region, the amygdala, which is involved in emotional and aggressive behavior [37].

## Materials and Methods

### Ethics Statement

All animal care and treatment was in compliance with, and all experiments approved by, the University of Virginia Animal Care and Use Committee.

### Animals

Animals were maintained on a 12:12 light/dark cycle (lights off at 1200, EST, for aggression; lights off at 1900, EST, for maternal behavior). Food (Harlan Teklad #7912) and water were provided *ad libitum*. To generate offspring for adult aggression experiments, DBA/2J (DBA) and C57BL/6J (B6) male and female mice (10 pairs of each strain) were purchased from Jackson Laboratories. To give all dams maternal experience, males and females were paired, mated, and males were removed prior to parturition (on average, after 14 days). Only females that successfully raised a litter to weaning were included in the study. Adult male (n = 15) and female (n = 15) B6 mice from our mouse colony were used as stimulus animals for social exposure tests. Intruders for aggression tests were age and size-matched A/J strain (an inbred line noted for its passivity) male mice, also from our mouse colony (n = 61). For maternal behavior experiments, adult male and multiparous female (n = 19) B6 mice from our breeding colony were paired to produce litters.

### Cross-Fostering

**Figure 1:**
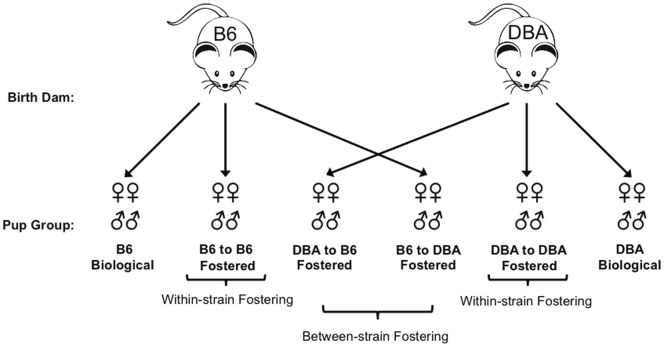
Illustration of fostering methods. Newborn B6 and DBA pups remained with their biological dam (n = 8 B6 and 12 DBA; Biological), were fostered to a dam of their own strain (n =  9 B6 and 6 DBA; Within-strain), or were reared by a dam of the other strain (n =  7 B6 and 15 DBA; Between-strain).

Experienced dams were paired with males, mating plugs were verified and, after 1–2 wks, males were removed. On the day of birth (PN0), pups were separated briefly (less than 5 minutes) from their dams, litters were culled to 2 males and 2 females, then pups were either returned to their biological dam (biological) or fostered to another dam (Figure 1). Between-strain fostering was conducted in both directions; B6 pups were fostered to DBA dams and vice-versa. Two additional groups of each mouse strain were fostered within-strain. Foster dams did not rear any of their own biological pups. On the day of weaning (PN21), mice were housed in same-sex sibling pairs. A total of 57 pups from 30 litters were used for this study, with 8 B6 and 12 DBA in the biological groups, 9 B6 and 6 DBA in the within-strain fostered groups, and 7 B6 and 15 DBA in the between-strain fostered groups.

### Surgery and Hormone Replacement

Because adult B6 males have lower levels of testosterone than DBA males [38] on PN60, offspring were bilaterally gonadectomized under isoflurane anesthesia as previously described [39], [40]. All mice received subcutaneous silastic implants (1.02 mm i.d., 2.16 mm o.d.) in the mid-scapular region filled to 10 mm in length with crystalline testosterone (T). In preliminary experiments, we determined that this dose of testosterone facilitated aggression in males of both strains, while maintaining the strain difference (data not shown). Mice were housed individually post-surgery for 2 weeks prior to aggression tests.

### Social Exposure and Aggression Testing

To enhance territorial behavior, cages were not changed for at least 5 days prior to aggression testing. Social exposure was conducted as described previously [39]. After 5 days of social exposure, mice were observed on 3 consecutive days of resident-intruder aggression testing as described previously [39], [41]. Gonad-intact male A/J mice were used as intruders, and the same intruder was used each day for each mouse. Intruders were placed into the cages and allowed to interact for up to 10 mins, or until 2 aggressive behaviors occurred. Behavioral measures were as defined previously [30]. The latency to display a combination of two aggressive behaviors (bite, chase, wrestle, or lunge) was recorded by direct observation (KHC). After an aggressive bout, intruders were removed to prevent injury and returned to their home-cages. Intruders were not used more than twice a day.

### Maternal Behavior

Biological (n = 9) and within-strain fostered (n = 10) litters were prepared as in Experiment 1. After culling litters, pups were scattered on the opposite side of the nest in the home-cage and dams were observed for 5 mins. Latencies to sniff the first pup, and to return each of the four pups to the nest were recorded. On the next day (PN1), dams were observed (by NLS) for two 60-minute periods in their home-cages. One observation was conducted during the light portion (at least 3 hrs before lights off) and one was conducted during the dark portion (at least 1 hr after lights off) of the light cycle. Within each 60-minute period, maternal behavior was scored every 20 seconds, resulting in a total of 360 observations for each dam. The following behavioral categories were recorded: (1) dam nursing pups in either high crouch or low crouch posture (2) dam licking and grooming pups, (3) dam building or rearranging nest, (4) dam on nest without nursing, licking and grooming, or nest-building, and (5) dam off nest.

### Tissue Collection and Quantitative Real-Time PCR

Approximately 48 hours after the final aggression test, adult B6 male offspring (n = 5–6 per group), from the biological and within-strain fostered groups were rapidly decapitated under isoflurane anesthesia, and brains were removed and frozen on dry ice. Using a cryostat, brains were then cut in coronal sections (120μm) onto slides. A tissue punch (1 mm) was used to dissect out the amygdala and the medial preoptic area (mPOA) following anatomical guidelines established by visually comparing slices to figures in the Mouse Brain Atlas [42]. The amygdala was collected in 2 bilateral punches from 8 sections corresponding to Atlas figures 33 through 40, and the mPOA was taken as one punch on the midline for 8 sections corresponding to Atlas figures 26 through 33.

Total RNA was isolated using a Qiagen RNeasy® Lipid Tissue Mini Kit. cDNA templates were generated with an ABI High Capacity cDNA Reverse Transcriptase Kit. An ABI StepOnePlus real-time PCR system was used to perform qPCR analysis with SYBR® Green or Taqman® reagents. Oligonucleotide primers were designed for *Avp*, *Avpr1a*, *Crh*, *Nr3c1*, *Ar* and *B2m*, while Taqman® probes were used for *Esr1* and *B2m* (Table 1). All gene transcription values were obtained from the StepOne™software and analyzed by the comparative ΔΔ cycle threshold (CT) method, using β2 microglobulin (*B2m*) as an endogenous control.

**Table 1:** List of quantitative PCR primers and Taqman® probes used for gene transcription analyses.

Primers:		
Gene	Forward primer	Reverse primer
***Avp***	TGCTCGCCAGGATGCTCAACAC	TTGCCGCCTCTTGGGCAGTT
***Avpr1a***	GCTGGCGGTGATTTTCGTG	GCAAACACCTGCAAGTGCT
***Crh***	GGAGAAGAGAGCGCCCCTAAC	TTCTTCACCCATGCGGATA
***Nr3c1***	GGATGCCATTATGGGGTCCT	TCGTTTTTCGAGCTTCCAGGT
***Ar***	AGAATCCCACATCCTGCTCAA	AAGTCCACGCTCACCATATGG
***B2m***	GGCTCACACTGAATTCACCCCCAC	ACATGTCTCGATCCCAGTAGACGGT

Probes:		
Gene	Identification Number	
***Esr1***	Mm00433149_m1	
***B2m***	Mm00437762_m1	

*Avp*, arginine vasopressin; *Avpr1a*, arginine vasopressin receptor 1A; *Crh*, corticotrophin releasing hormone; *Nr3c1*, glucocorticoid receptor; *Ar*, androgen receptor; *B2m*, β2 microglobulin; *Esr1*, estrogen receptor α.

### Data Analysis

Aggression latencies were analyzed via repeated measures ANOVA as previously described [39], [43], with pup strain and fostering groups as variables, and paired comparisons were made with Fisher's LSD post-tests. Percentage of animals attacking was analyzed using Fisher's Exact tests and Bonferroni t-tests corrected for multiple comparisons. Gene transcription data were analyzed with Pearson product-moment correlations. For pup retrieval data, non-cumulative latencies were analyzed by repeated measures ANOVA with fostering group as the independent variable and the number of pup (in order of retrieval) as the within-factor. Home-cage maternal behavior was analyzed using Bonferroni t-tests corrected for multiple comparisons.

## Results

### Fostering increases aggressive behavior in B6 male mice

**Figure 2:**
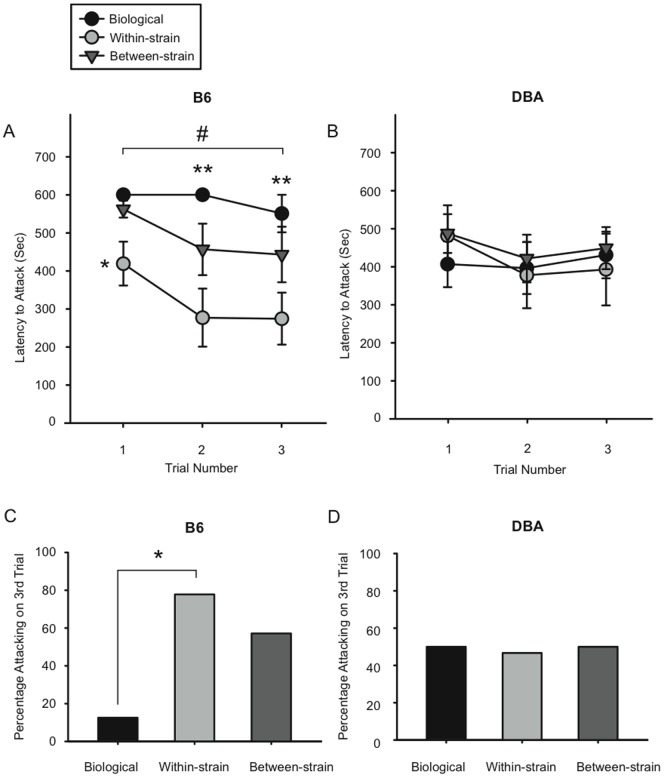
Fostering influences aggressive behavior in B6 male mice. (A, B) Mean (+/- SEM) attack latencies (in seconds) on each aggression trial for B6 (A) and DBA (B) male offspring. B6 males reared by their biological dams took significantly longer to attack intruders than within-strain and between-strain fostered males (p<0.0002). * Within-strain fostered B6 males had significantly shorter attack latencies than those reared by biological dams on the first trial. ** B6 males reared by biological dams had significantly longer attack latencies than within-strain and between-strain fostered B6 males on the second and third trials. # Latencies to attack on the first trial were longer than those on the second and third trials (p<0.004). (C, D) Percentage of individuals attacking on the third aggression test for B6 (C) and DBA (D) male offspring. * Percentage of attacking males was significantly higher in the within-strain fostered B6 group than the biological B6 group (p = 0.0304).

Using both within-strain and between-strain fostering (Figure 1), we tested the hypothesis that differences in aggression between inbred mouse strains are influenced by differences in maternal behavior. We found a significant interaction between strain and foster dam on aggressive behavior (F(2,170) =  4.16; p<0.03). When B6 and DBA males were analyzed separately, we noted a main effect of rearing dam on the latency of attack in B6 males, with males reared by biological dams taking significantly longer to attack intruders than males that were within-strain or between-strain fostered (F(2,41) = 8.99, p<0.002; Figure 2A). Fisher's LSD post-tests revealed that B6 males reared by biological dams had significantly longer attack latencies than B6 within-strain fostered pups on all three trials, and significantly longer latencies than B6 between-strain fostered males on trials two and three. There was also a main effect of trial; latencies on the first trial were longer than those on trials two or three (F(2,41) = 6.39, p<0.004; Figure 2A). Interestingly, the effect of fostering on attack latency was specific to B6 males. DBA males from all three groups showed similar attack latencies on each trial, but were significantly more aggressive than the B6 biological group, overall (F(1,60) = 5.91, p<0.03; Figure 2B). Moreover, the percentage of individuals attacking on the third aggression test was significantly higher for B6 within-strain fostered males than B6 males reared by biological dams (p = 0.0304), Figure 2C). Taken together, these results suggest that fostering, in and of itself, alters the aggressive behaviors of male B6 mice, while having no effect on male DBA mice.

### Foster pups change maternal behavior

**Figure 3:**
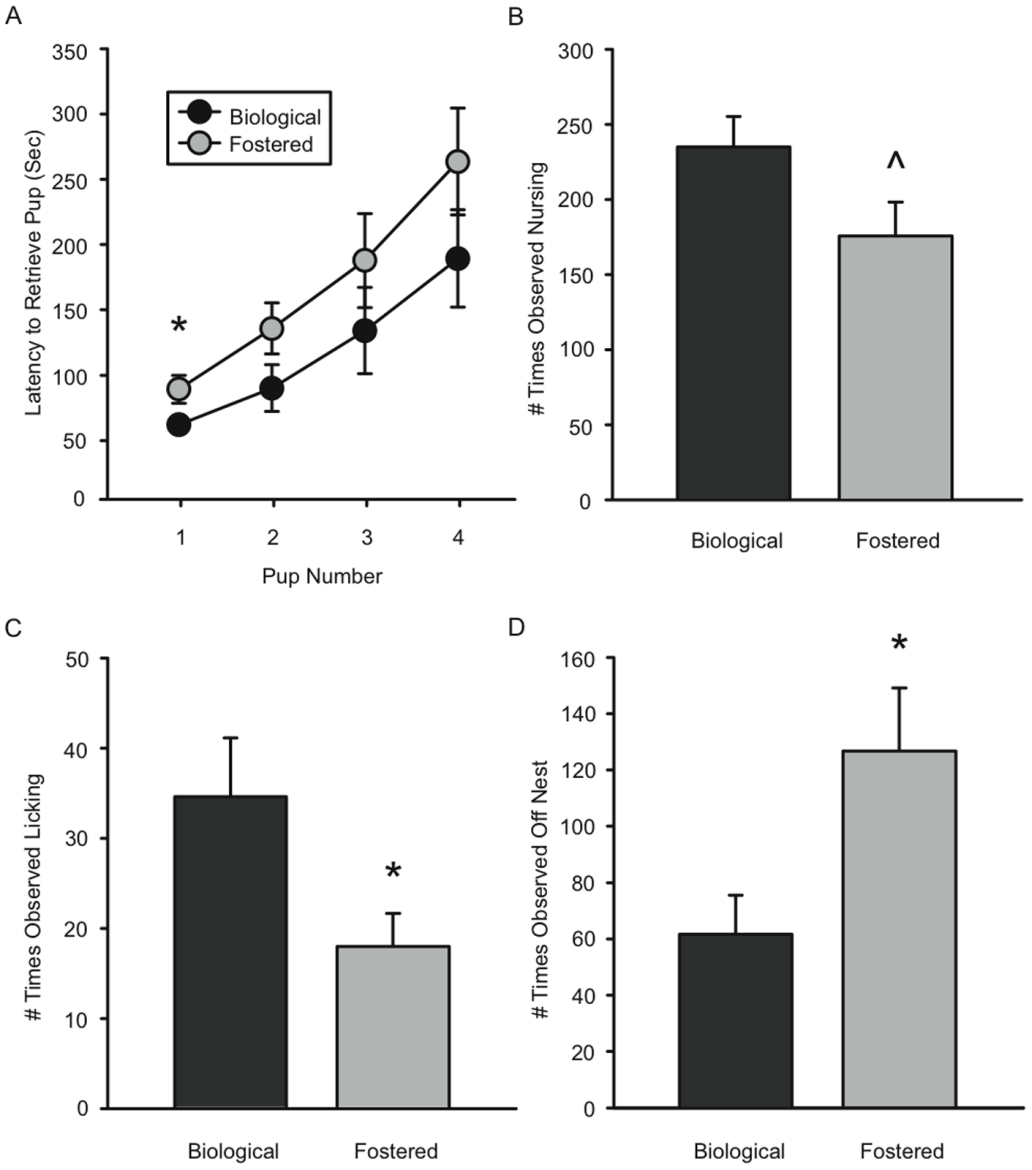
Foster pups change maternal behavior of B6 dams. (A) Mean (+/– SEM) cumulative latencies to retrieve each of 4 pups to the nest. * Fostering significantly increased the latency to retrieve pups in the home-cage (p<0.05). Specifically, foster dams (n = 10) were significantly slower than biological dams (n = 9) to retrieve the first pup (p<0.04). (B–D) Mean (+/– SEM) number of times dams were seen nursing (B), licking and grooming pups (C), and off the nest (D) * Foster dams licked and groomed their pups significantly less than biological dams (p<0.04) and were off the nest more frequently than biological dams (p<0.04). ^∧^ Foster dams exhibited a trend to nurse less frequently than biological dams (p<0.08).

To ask if maternal behavior was affected by within-strain fostering, we fostered B6 pups to same-strain dams and measured maternal behavior of biological versus fostered dams. Intriguingly, pups reared by their own mothers were treated differently than foster pups. There was a significant effect of fostering on latency to retrieve pups, with foster dams taking longer to retrieve pups back to the nest (F(1,18) = 4.58, p<0.05; Figure 3A). Specifically, planned comparisons revealed that foster dams took significantly longer to retrieve the first pup than did biological dams (p<0.04). In addition to retrieval differences, in home-cage behavioral observations, biological dams spent more time licking their pups (p<0.04), and less time off the nest (p<0.04) than foster dams (Figure 3C,D). There was a trend for biological dams to spend more time than foster dams engaged in high or low arched back nursing (p<0.08; Figure 3B). Taken together, these results suggest that maternal behavior was affected by the identity of the pups: biological or fostered.

### Differences in maternal rearing impact mRNA in the amygdala

Lastly, we examined whether differences in maternal environment had effects on gene transcription in offspring. We also determined if these differences in gene transcription correlated with individual aggressive behavior. We examined gene transcription in the amygdalae of within-strain fostered and biologically reared B6 male mice collected approximately 48 hours after the final aggression test. While *Avp* and *Crh* mRNA levels may respond acutely to aggressive encounters, studies on short-term responses of AVP release [44] and *Crh* mRNA [45] show that these changes are unlikely to persist for more than 48 hours. Therefore, the observed gene transcription levels likely reflect that of basal conditions.

**Figure 4:**
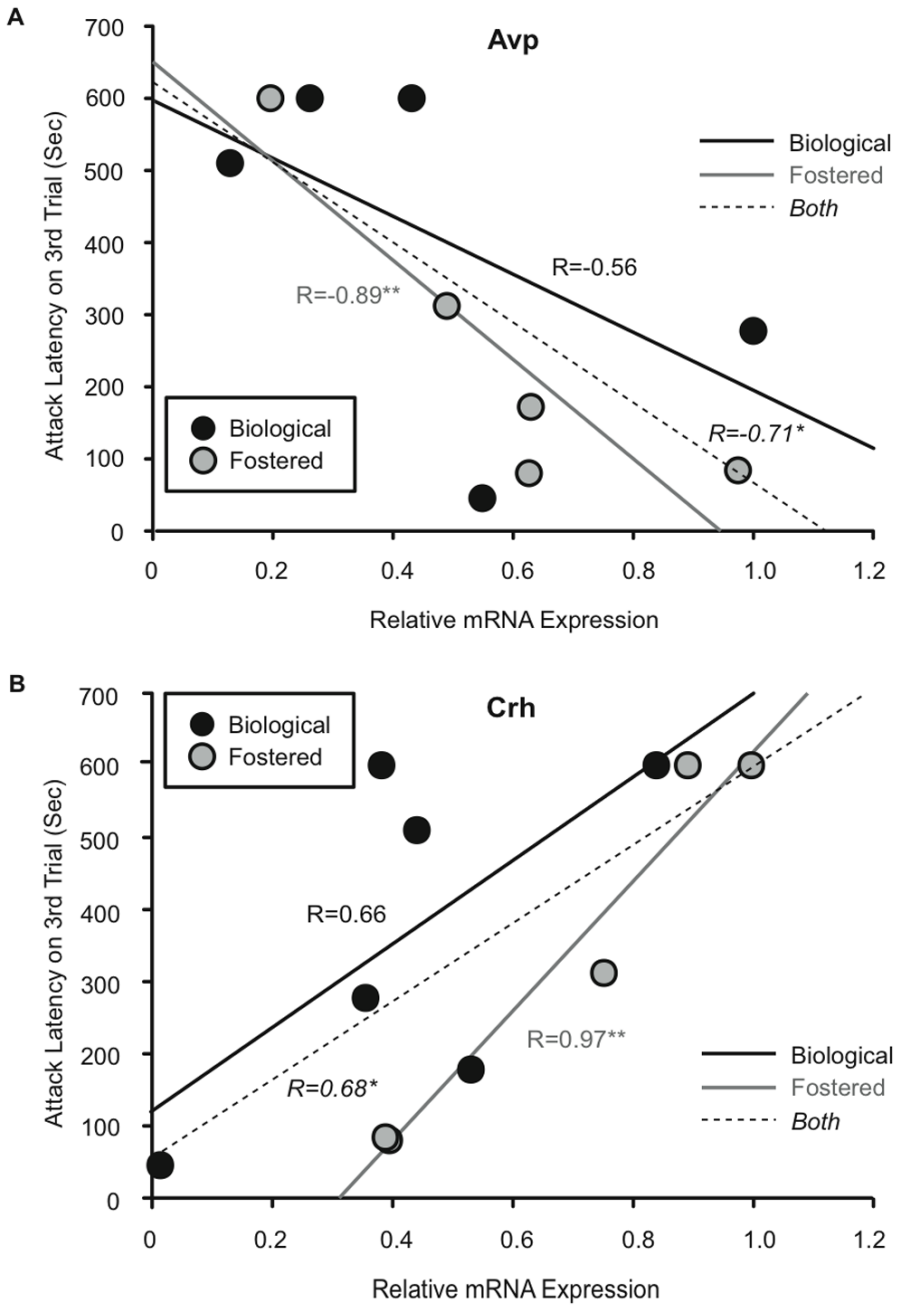
Amygdala gene transcription is altered in fostered B6 male offspring. Scatter plots with best-fit regression lines showing correlations between relative gene expression and latency to attack in the third aggression test of biological (n = 5) or fostered (n = 6) male B6 offspring. (A) * Attack latency correlated negatively with *Avp* mRNA transcription (r = –0.71, p<0.03; dashed line). ** When separated by group, attack latency correlated negatively with *Avp* mRNA transcription in fostered (r =  –0.89, p<0.05; gray solid line) but not biological (r = –0.56, p = 0.33; black solid line) offspring. (B) * Attack latency correlated positively with Crh mRNA transcription (r = 0.68, p<0.03; dashed line). ** When separated by group, attack latency correlated positively with *Crh* mRNA transcription in fostered (r = 0.97, p<0.005; gray solid line) but not biological (r = 0.66, p = 0.15; black solid line) offspring.

We found that longer average attack latencies (across all three trials) correlated with increased *Crh* gene transcription (r = 0.61, p<0.05) while there was no significant correlation with *Avp* gene expression (r = –0.46, p = 0.18) in the amygdala. However, longer attack latencies in the third and final aggression test were significantly correlated with both increased *Crh* gene transcription (r = 0.68, p<0.03; Figure 4B) and decreased *Avp* gene transcription (r = –0.71, p<0.03; Figure 4A) in the amygdala. When transcription of these genes in brains of biological and foster males was analyzed separately (Figure 4A,B), it was clear that the correlations were driven by the fostered males. There was a trend for average attack latency of fostered males to be negatively correlated with *Avp* gene transcription (r = –0.88, p = 0.051), and a significant positive correlation with *Crh* gene transcription (r = 0.98, p<0.01). Attack latencies in the final aggression test followed the same pattern (Figure 4A (r = –0.89, p<0.05 for *Avp* and Figure 4B r = 0.97, p<0.005 for *Crh*). We speculate that changes in aggressive behavior in fostered males were mediated through these genes. None of the other candidate genes examined showed significant differences between groups or correlations with aggressive behavior in biological or fostered offspring (Table 2).

**Table 2:** Correlation coefficients and p-values for relative gene expression and attack latency in the 3^rd^ aggression trial for biologically reared (biological) and within-strain fostered (fostered) B6 males.

		Biological		Fostered	
Region	Gene	r	p	r	p
AMYG	*Avpr1A*	0.25	0.63	–0.01	0.99
	*Esr1*	0.05	0.91	–0.24	0.70
	*Nr3c1*	–0.23	0.66	–0.42	0.41
mPOA	*Avpr1a*	0.26	0.62	–0.09	0.87
	*Esr1*	–0.09	0.87	–0.27	0.61
	*Ar*	–0.39	0.45	–0.44	0.38

AMYG, amygdala; mPOA, medial preoptic area; r, Pearson correlation coefficient; *Avpr1a*, arginine vasopressin receptor 1A; *Nr3c1*, glucocorticoid receptor; *Ar*, androgen receptor; *B2m*, β2 microglobulin; *Esr1*, estrogen receptor α.

## Discussion and Conclusions

These studies show that rearing by a non-biological dam, whether it is by a female of the same inbred strain or another strain, increases aggressive behavior in B6 male mice. Our data brings to the foreground the impact of within-strain fostering on social behavior, as fostered B6 males, regardless of the strain of the foster dam, displayed increased aggressive behavior as compared to mice reared by biological dams. To our knowledge, only one set of studies has tested the effects of fostering on aggression using inbred laboratory mouse strains (ABG and AB//Halle; [46]), although several studies have investigated maternal influences on aggression in wild or outbred mice [18], [34], [35], [36]. Utilizing cross fostering between two species (one aggressive, one relatively non-aggressive) of *Peromyscus* mice, Bester-Meredith and colleagues (2001) found that aggression in fostered males matched levels in foster parents, indicating that maternal-infant interactions play a role in determining adult aggressive behavior. In contrast, a within-strain fostering experiment using outbred CD-1 mice showed no effect of fostering on aggressive behavior [47]; and, in a cross-fostering experiment using two wild-derived strains of inbred mice selected for different levels of aggression, there was no effect of either within- or between-strain fostering on aggressive behavior [48], suggesting that environmental factors are less important in mice bred selectively for this trait. Our results reinforce these earlier studies by showing that the contributions of genetics and postnatal environment on aggressive behavior depend on the species or strain of mouse.

Whereas fostering had an effect on the behavior of B6 males, it had no such effect on DBA males, demonstrating that different mouse strains with different genetic background and prenatal environment do not respond identically to changes in the maternal environment. Our findings are similar to one prior study, which, using a similar fostering paradigm, showed an effect of both within and between-strain fostering on open field activity in B6, but not DBA, mice [22]. Strain-specific effects in the reverse direction have also been seen in depressive-like behavior, which is altered in response to maternal separation in DBA but not B6 animals [49]. Although we did not pursue further studies with the DBA strain, either genetic differences, or differences in the prenatal environment of B6 and DBA mice, or both factors, likely contribute to their differential response to environmental manipulations. DBA males may be more resilient to the effects of fostering or other early-life stress; alternatively, it may be that the DBA dams’ maternal behavior is less sensitive to fostering than B6 dams. It would be worthwhile to further investigate possible genetic factors, or, using embryo transfer, the prenatal factors [50] that underlie these strain differences using DBA/B6 hybrid mice.

Maternal fostering can influence a variety of other phenotypes in offspring. For example, between-strain fostering alters anxiety behaviors [51], autoimmune disease [52], and body weight [48]. Importantly, within-strain fostering also influences anxiety, juvenile social behaviors [22], [53], [54], and body weight [47], [55]. Taken together with the data shown here, we propose that fostering itself alters the maternal environment and mediates permanent changes in offspring phenotypes. In fact, when examining the percentage of males attacking, it appears that within-strain fostering may have an even larger effect on male aggression than fostering between strains. Accordingly, we found that maternal behavior of B6 foster dams was significantly different from behavior of dams rearing their biological litters. Foster dams were slower to retrieve non-biological pups, spent more time off the nest, and less time licking pups as compared to biological dams. These data indicate that B6 dams can discriminate between biological and fostered offspring and alter their maternal care accordingly. Sensory and/or behavioral differences in pups from a different dam likely provoke the differences in maternal care. While we did not measure any pup behaviors, such as ultrasonic vocalizations, which may be different in fostered pups and/or pups of different strains [56], [57], it is likely that the behavior of pups contributed to these differences in maternal behavior. However, our results are consistent with other studies on maternal recognition of biological versus non-biological young in out-bred mice [58] and studies that have shown alterations in maternal behavior with between-strain fostering [17], [20], [21], [59] and within-strain fostering [21], [59]. Notably, despite the implication that the maternal environment changes adult phenotypes in offspring, only a few studies have directly examined maternal behavior of foster dams in conjunction with altered phenotypes of offspring [47], [51], and none report differences in maternal behavior with fostering in association with changes in offspring behavior. Thus, our study makes an important contribution to the understanding of maternal behavior in relation to fostering and offspring aggressive behavior.

Maternal care is able to influence offspring behaviors via modulation of gene transcription (reviewed in [13]). Here we found that the transcription of corticotrophin releasing hormone (*Crh*) and arginine vasopressin (*Avp*) in the amygdalae of male mice correlated with their attack latencies. However, only fostered males showed this correlation, suggesting that fostering may increase aggression by acting on brain circuits involving *Crh* and *Avp* production in the amygdala. Although genes involved in aggressive behavior have not been studied within in the context of variations in maternal care, maternal separation, a form of early post-natal stress, enhances the display of aggression in male rats [5], [6], and this increase in aggressiveness correlates with higher *Avp* mRNA transcription in the anterior hypothalamus [5]. Consistent with these results, we found that increased *Avp* mRNA transcription correlates with higher level of aggression in fostered B6 males in the amygdala, a region in which local synthesis and release of vasopressin are important for aggressive behavior [60], [61]. In addition, increased maternal licking and grooming led to more vasopressin 1A receptor (V1aR) binding in the amygdala of adult male rats [62]. While we did not examine receptor binding in our study, nor did we observe any effects of fostering on V1aR gene transcription in either the amygdala or the POA. In B6 mice, maternal separation is associated with long-term hypomethylation of the *Avp* enhancer [11], constituting a mechanism through which *Avp* mRNA can become elevated in adult offspring subjected to maternal separation. Therefore, our finding that decreased maternal behavior led to altered vasopressin in the amygdala and increased aggression in fostered offspring, while correlational, fits within the framework of what is understood about maternal influences on vasopressin, and may be mediated by altered methylation of the *Avp* gene.

Previous studies have shown that variations in the early-life environment can also affect HPA-mediated neuroendocrine and behavioral responses through altering components of the glucocorticoid feedback mechanism and *Crh* mRNA in the hypothalamus [7]. We found that fostering altered the amount of *Crh* mRNA transcription the amygdala, a region important for behavioral responses to stress and anxiety-related behavior [63], [64]. Notably, CRH knockdown in the central amygdala decreases anxiety, suggesting that CRH in the amygdala positively regulates anxiety [64]. Given our findings that *Crh* mRNA was altered in fostered B6 male offspring and that fostering led to higher levels of aggression, it would be worthwhile to examine the effect of fostering on the relationship between anxiety-related behavior and aggression in future studies. Interestingly, rats selectively bred for low anxiety behavior exhibit significantly higher aggression than non-selectively bred rats [6], and anxiety and aggression share neurochemical and neuroanatomical networks [12].

In summary, this is the first study to find effects of within-strain and between-strain fostering on aggressive behavior in an inbred mouse strain, while also showing that within-strain fostering has a long-lasting impact on *Avp* and *Crh* mRNA in the amygdala, a brain region important for aggressive behavior. Moreover, we show that these effects are likely mediated by differences in maternal behavior in B6 mouse dams towards fostered and biological pups. These findings have broader implications for experimental design and interpretation of results in studies using fostering protocols. While, at times, fostering is necessary to prevent off-target effects of drugs or other independent variables, it may also be necessary to include a biologically-reared group in these studies and/or evaluate maternal behavior to assess the contributions of the maternal environment to measured phenotypes.
